# Forehead or ear temperature measurement cannot replace rectal measurements, except for screening purposes

**DOI:** 10.1186/s12887-018-0994-1

**Published:** 2018-01-26

**Authors:** Christian Backer Mogensen, Lena Wittenhoff, Gitte Fruerhøj, Stephen Hansen

**Affiliations:** 1 University of Southern Denmark, Institute for Regional Health Research, Kresten Philipsensvej 15, 6200 Aabenraa, Denmark; 2 Pediatric Department, Hospital of Southern Denmark, Aabenraa, Denmark

**Keywords:** Pediatric, Temperature measurement, Ear temperature, Tympanic temperature, Rectal temperature, Temporal temperature, Emergency care

## Abstract

**Background:**

Measuring rectal temperature in children is the gold standard, but ear or forehead measures are less traumatic and faster. The quality of non-invasive devices has improved but concerns remain whether they are reliable enough to substitute rectal thermometers.

The aim was to evaluate in a real-life children population whether the forehead or ear temperature measurements could be used in screening to detect fever and if the agreement with the rectal temperature for different age groups is acceptable for clinical use.

**Methods:**

Cross-sectional clinical study comparing temporal and tympanic temperatures to rectal temperature in 0–18-year-old children. The ear thermometer was a Pro 4000 Thermoscan, the temporal Exergen TAT. Rectal temperature ≥ 38.0 °C was defined as fever.

**Results:**

Among 995 children, 39% had a fever. The ear thermometer had a significantly greater ability to detect fever than the temporal thermometer (AUC 0.972; 95% CI: 0.963–0.981 versus AUC 0.931; 95% CI: 0.915–0.947, *p* < 0.0001). Both devices had the lowest sensitivity in the youngest and oldest children, and only the ear thermometer reached a sensitivity above 90% in the 0.5–5-year age group. The Bland-Altman analysis showed that the 95% limits of agreement for the temporal thermometer was between − 1.2 to + 1.5 °C and for the ear thermometer between − 0.97 to + 1.07 °C.

**Conclusions:**

Based on a large sample of children, the temporal measurement of temperature is not currently recommendable, but with the technology used in this study the ear measurement proved useful for screening purposes, especially among children aged 6 months to 5 years. For the exact measurement of temperature, the rectal method is still recommended.

**Electronic supplementary material:**

The online version of this article (10.1186/s12887-018-0994-1) contains supplementary material, which is available to authorized users.

## Background

Measuring the temperature in children with acute conditions is essential. Rectal measurement is the gold standard, [1] but there are alternatives. Using the ear or forehead temperature is less traumatic and allows a faster triage. A recent meta-analysis based on 75 studies concluded, that high quality data were limited and peripheral thermometers did not have clinically acceptable accuracy to be recommended for clinical use. Among these studies 53% were more than 10 years old [2].

However, the quality of these devices for rapid, non-invasive temperature measurements has improved in recent years and suggestions have been made to replace rectal thermometers with these alternatives for children or to use the devices as screening instruments for fever [1, 3]. A range of studies support this suggestion [4–7]. Since these studies have limitations in size or methodology, however, concerns have been raised as to whether the new devices are reliable enough to substitute for the rectal thermometer and if this reliability is consistent within different age groups [8].

The aim of the present study was, in a real-life population of children, acutely referred to a pediatric department; firstly, to evaluate, based on current technology, whether forehead or ear temperature measurements could be used as a screening tool to detect fever; and, secondly, to evaluate how well these measurements agrees with the rectal temperature within different age groups of children.

## Methods

We performed a cross-sectional clinical study, comparing the temporal and tympanic temperatures to the rectal temperature in 0–18-year-old children in real-life assessments. Children who were referred for any acute condition by the general practitioner to the pediatric emergency department of the hospital of Southern Jutland were included if oral parental consent was obtained and time allowed. We did not include children who were admitted directly to the neonatal ward, who had anal atresia or deformities of the outer ear. The three temperature measurements were performed immediately after each other by the same nurse in the following order: temporal, tympanic, and rectal measurement. The temperatures were recorded immediately by the nurse to pre-printed files together with information about gender and age in months. The new generation of tympanic thermometers measures the temperature over a wider angle and uses multiple measuring points, compared to former devices. We chose a Braun Welch Allyn Pro 4000 Thermoscan, released in 2006, where a heating element in the sensor warms the probe tip to just under normal body temperature to avoid cooling the ear canal [9]. The temporal thermometer was an infrared non-invasive Exergen TAT from GE Healthcare. The probe of the device is gently positioned on the center of the forehead and lightly slid across the forehead, keeping the sensor in contact with the skin until the hairline is reached while the temporal artery temperature is measured. The rectal thermometer was an OMRON Healthcare, maximum thermometer. All of the thermometers were purchased in 2010 for this study only. All of the nurses in the pediatric department were instructed in the correct use of the three devices. Compliance with the instructions was intermittently verified by the study nurse.

The results were intended to be analyzed as a total population and in six different age groups. We aimed at a sample size for analysis of minimum 99 children within each of the six age groups to be able to detect a minimum mean difference of +/− 0.3 °C with a standard deviation of 0.65 °C between the rectal and the alternative measurement, a 90% power, and a significance level of 0.05, assuming normal distribution of the temperatures. The obtained data were normal distributed and the standard deviation around 1 °C. Further details are reported in Additional file 1.

The recorded results were transferred to and analyzed in Stata 14. The rectal measurement was the reference temperature. A temperature of ≥38.0 °C was defined as fever. The ability to be used as a screening tool was evaluated by calculating the sensitivity, specificity, predictive values, and correct classification with a 95% confidence interval (CI). We also calculated the area under the curve (AUC) in a receiver–operator characteristic (ROC) diagram, with a threshold for classification boundary of fever to 38.0 °C rectally. Since a device used for screening for fever should have a high sensitivity in order not to classify a febrile patient as not febrile, we sought a cut point for the examined devices where 95% of all children with rectal temperature ≥ 38.0 °C would be detected.

The agreement of the non-rectal thermometers with the rectal temperature was examined in a Bland-Altman analysis and plots to determine the level of agreement and the 95% limits of agreement.

The rectal temperature was the standard temperature measurement used for all children in the department, but was supplied by an initial ear- or temporal measurement as a screening for fever on arrival. The study was considered a quality assurance study of already implemented routines and no ethical clearance was required. Oral consent was obtained from the parents before the temperature measurements were performed. All of the data was anonymously recorded and the Danish Data Protection Agency confirmed that registration of the database was unnecessary.

## Results

During the period under study, from December 2010 to November 2011, 2523 children were referred to the pediatric department. Among these children, 996 parents were asked to allow their child to participate, all of whom consented. Due to technical problems, the thermometers were unable to measure the temperature in 30 forehead measurements and 1 rectal temperature measurement, which left 995 children with a reference temperature and at least one other measurement for further analysis.

The median age of the children was 24 months (Inter Quartile Range 11–70 months) with 489 (49%) girls, and the rectal temperatures indicated that 39% of the children had a fever.

**Fig. 1:**
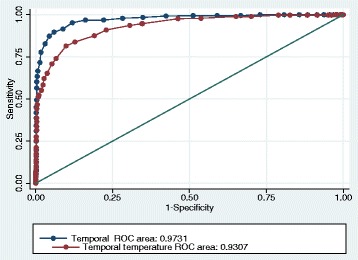
Temporal and tympanic measurements for fever ≥38 °C

Table 1 shows the screening measures for the detection of fever using the temporal or tympanic thermometer. For the whole population, the tympanic thermometer had a significantly greater ability to detect fever than the temporal thermometer (AUC 0.972; 95% CI: 0.963–0.981 versus AUC 0.931; 95% CI: 0.915–0.947, *p* < 0.0001), Fig. 1. The temporal thermometer had a sensitivity ranging from 72 to 92%, while the tympanic thermometer ranged from 67 to 93% in sensitivity in the different age groups. Both devices had the lowest sensitivity in the youngest and oldest children, and only the tympanic thermometer reached a sensitivity above 90% in the group of children aged 6 months to 5 years. Using a cut point of 37.4 °C for the temporal measurements, which 66% of all children fulfilled, resulted in a sensitivity for fever of > 95% and a positive predictive value of 57%. For the tympanic measurements the cut point for 95% sensitivity was 37.8 °C, which 44% of the children fulfilled with a positive predictive value of 83%.

**Table 1:** Temporal and tympanic measurements of temperature as a screening tool for fever (> = 38.0 °C)

			Screening measures (in %)											
Age group	Method	No. of children	Sensitivity	95% CI	Specificity	95% CI	Positive predictive value	95% CI	Negative predictive value	95% CI	Correctly classified	95% CI	AUC	95% CI
Total	Temporal	965	81	77-85	90	87-92	84	80-88	88	86-91	87	84-89	0.931	0.915-0.947
	Tympanic	995	89	86-92	94	92-96	91	87-93	93	91-95	92	90-94	0.972	0.963-0.981
0-5 months	Temporal	133	75	53-90	93	86-97	69	48-86	94	88-98	89	83-94	0.924	0.875-0.973
	Tympanic	136	67	45-84	96	91-99	80	56-94	93	87-97	91	85-95	0.936	0.875-0.996
6-11 months	Temporal	124	80	67-89	91	82-97	88	75-95	85	75-92	86	79-92	0.943	0.905-0.980
	Tympanic	128	93	82-98	96	89-99	94	84-99	95	87-99	95	89-98	0.984	0.964-1.000
12-35 months	Temporal	311	82	75-88	87	81-92	87	81-92	82	75-88	85	80-88	0.919	0.889-0.949
	Tympanic	320	92	86-95	93	88-96	93	88-97	91	86-95	91	89-95	0.969	0.951-0.987
3-5 years	Temporal	162	92	83-97	88	79-94	86	76-93	93	85-97	90	85-94	0.961	0.934-0.986
	Tympanic	169	92	84-97	90	82-96	89	80-95	93	86-98	91	86-95	0.981	0.964-0.997
6-11 years	Temporal	131	72	56-85	91	83-96	80	64-91	87	78-93	85	77-90	0.900	0.842-0.958
	Tympanic	135	87	74-95	92	85-97	85	72-94	93	86-98	90	84-95	0.968	0.943-0.993
12-18 years	Temporal	104	74	52-90	93	85-97	74	52-90	93	85-97	88	80-94	0.932	0.878-0.986
	Tympanic	107	84	64-96	98	92-100	91	72-99	95	88-99	94	88-98	0.963	0.925-1.000

*CI* confidence interval, *AUC* area under receiver-operating characteristics curve

Table 2 reports the mean temperatures and the Bland-Altman comparison in the age groups measured by the different devices.

**Table 2:** Temperature variation within the age groups

					Bland-Altman comparison (°C)		
Age group	No. of children	Method	Mean temperature (°C)	95% CI	Mean difference	95% CI	95% prediction interval
Total	965	Temporal	37.9	37.9-38.0	0.13	0.08-0.17	−1.20 - 1.49
	995	Tympanic	37.8	37.8-37.9	0.05	0.02- 0.08	−0.97 - 1.07
	995	Rectal	37.8	37.7-37.9	reference		
0-5 months	133	Temporal	37.5	37.4-37.7	0.16	0.06 - 0.25	- 0.97 - 1.29
	136	Tympanic	37.1	37.1-37.3	−0.14	− 0.23 - -0.06	−1.15 - 0.87
	136	Rectal	37.4	37.2-37.5	reference		
6-11 months	124	Temporal	37.9	37.7-38.1	−0.04	−0.16 - 0.09	−1.43 - 1.36
	128	Tympanic	38.0	37.8-38.1	0.00	−0.08 - 0.08	−0.89 - 0.90
	128	Rectal	38.0	37.8-38.1	reference		
12-35 months	311	Temporal	38.1	38.0-38.3	0.06	−0.02 - 0.14	−1.35 - 1.46
	320	Tympanic	38.1	38.0-38.2	0.01	−0.04 - 0.07	−1.01 - 1.02
	320	Rectal	38.1	37.9-38.2	reference		
3-5 years	162	Temporal	38.2	38.0-38.3	0.25	0.16 - 0.35	0.96 - 1.47
	169	Tympanic	38.1	37.9-38.3	0.19	0.12 - 0.26	−0.73 - 1.11
	169	Rectal	37.9	37.7-38.1	reference		
6-11 years	131	Temporal	37.8	37.7-38.0	0.21	0.10 - 0.32	−1.06 - 1.49
	135	Tympanic	37.8	37.6-38.0	0.18	0.09 - 0.27	−0.85 - 1.21
	135	Rectal	37.6	37.5-37.8	reference		
12-18 years	104	Temporal	37.6	37.4-37.8	0.17	0.04 - 0.30	−1.18 - 1.52
	107	Tympanic	37.5	37.3-37.7	0.05	−0.06 - 0.16	−1.07 - 1.17
	107	Rectal	37.4	37.2-37.6	reference		

**Fig. 2:**
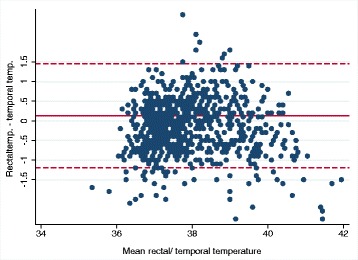
Bland-Altman plot for temporal and rectal temperatures

The mean difference from the temporal to the rectal temperature was small but significant: 0.13 °C (95% CI 0.08–0.17 °C, *p* < 0.0001). In the different age groups, the mean difference ranged from − 0.04 to 0.25 °C. The 95% limits of agreement for the temporal thermometer was between − 1.2 to + 1.5 °C, and the same variation was seen within the different age groups. Figure 2 indicates a systematic difference for higher temporal temperatures.

**Fig. 3:**
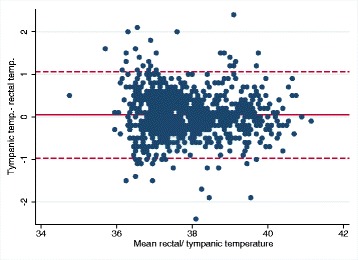
Bland-Altman plot for tympanic and rectal temperatures

The mean difference from the tympanic to the rectal temperature was 0.05 °C (95% CI 0.02–0.08 °C, p: 0.004), and varied between − 0.14 °C to 0.19 °C in the age groups. The 95% limits of agreement for the tympanic thermometer was between − 0.97 to + 1.07 °C for the whole group and almost the same interval within the different age groups. Figure 3 does not indicate any systematic differences.

## Discussion

We found, that as a screening tool to detect fever ≥38.0 °C, the tympanic thermometer was significantly better than the temporal thermometer. However, the 6 months to 5 years age group was the only one in which the sensitivity of the ear thermometer was above 90%. If the tympanic device was used as a screening tool for fever and the tympanic measurement ≥37.8 °C was regarded as a positive test of fever, this meant that more than 95% of all febrile children would be detected at the screening, that only 44% of the children needed to continue with a rectal measurement and among these children 83% would be truly febrile. The forehead and ear temperature measurements were both poor to estimate the exact rectal temperature, with a 95% limits of agreement from around − 1.5 to + 1.2 °C for the forehead measurements and − 1 to + 1 °C for the ear measurements.

The search for alternatives to a rectal measurement of the temperature in children has been going on for decades. Several reviews have reported disappointing results [2, 10, 11]. The instruments used in our study were based on current technologies and developed for professional use. These devices have now been evaluated in a range of studies with mixed results.

The temporal device, TAT-5000 IR, was found to be reliable in children in studies from the US of 47 children, India 50 children, and Turkey with 218 children. These studies only found minor temperature differences to the rectal temperature and sensitivities above 80% to detect fever [4–6].

In contrast, other research groups found that the temporal device was still too inaccurate to be recommended. In Belgium, 294 children had a deviation from the rectal temperature from − 1.32 to + 1.33 °C and the thermometer had sensitivity of 41% to detect fever [8]. Four studies from the US of 44, 52, 147, and 474 children found too large mean differences in temperature and sensitivities from 53 to 70% to detect fever, [12–15] which are similar to findings among 156 children from Nigeria and 205 children from Argentina [16, 17]. These studies are weakened by small numbers, inadequate statistical methods or no sub-analysis within age groups. Our study does not have these limitations and concludes that temporal measurement is not able to measure the temperature within a clinically safe limits of agreement range nor is it useful as a screening tool for fever.

The infrared ear thermometer, ThermoScan PRO 4000, was examined in a single study of 205 children from Argentina, which also compared it against the same temporal device as in our study: the TAT-5000 IR. The mean temperature difference to rectal temperature was 0.001 °C and the 95% limits of agreement from − 0.77 to + 0.77 °C. The sensitivity to detect fever was 92% [17]. These findings are quite similar to our findings. We conclude that the tympanic thermometer is better than the temporal thermometer at measuring temperature but still has a wide limit of agreement range, and almost every tenth child with a fever will go undetected using this method.

These conclusions are valid for the devices used in the study. However, a recent study from Switzerland reached similar results, using other devices which were based on the same technologies [18].

Our findings have a number of clinical implications. Firstly, temporal devices have no place as a substitute or screening tool for temperature measurements. Secondly, the tympanic thermometer has reached a level of accuracy where it can be used as a screening tool for detecting fever, with an AUC of 0.97 and more than 95% of all children with fever will be detected if the cut point for tympanic measurement is 37.8 °C. It should be considered, however, that there will still be 5% of the children who are not detected. This means, that an approach of screening all children with the tympanic device and continue with a rectal measurement if the screening is positive would result in that less than half of the children would need a rectal measurement. Thirdly, the usefulness of tympanic temperature to measure the exact temperature is less convincing and the clinicians must accept a limits of agreement range of around +/− 1 °C, which is too wide for clinical use, where a range of less than +/− 0.5 °C has been considered acceptable [13].

The strength of the current study is that it is a comprehensive real-life pragmatic study with sub-analyses in different age groups. Furthermore, in contrast to most of the published studies, it was analyzed with appropriate statistical methods, including Bland-Altman analysis [19, 20]. The study is weakened by the fact that the patients were not consecutively included, only when time allowed. Only 39% had fever, which limits the analysis, even though this is partially compensated by the high number of participants. Since the study was performed anonymously, no comparison was possible between the participating and non-participating children to assess selection bias. Furthermore, the order of temperature measurements was the same throughout the study: temporal, tympanic and rectal. We did so to reduce the risk of crying which could influence the temporal measurements. The procedure might introduce a bias however, if the temperature lowered during the examination. We tried to avoid this by undressing the child partially and only for a short time after the two other measurements had been performed. Finally, in accordance with the vast majority of other studies, and since our clinical guidelines and everyday clinical practice base on rectal measurements, we chose the rectal temperature as the gold standard to represent the core temperature. It has been questioned whether the rectal temperature represents the true core temperature better than the tympanic temperature [1]. Our study design does not address this question, instead merely examining whether the alternatives could replace the well-established rectal method.

The examined ear device is useful for screening purposes, but there is still room for improvement. We encourage the continued search for and development of new methods and technologies to replace the inconvenience of the rectal thermometer. For each new device developed, it is crucial to evaluate the accuracy in a clinical setting by using sufficient numbers of patients and appropriate methods for comparison.

## Conclusion

Based on a large sample of children, we found that temporal measurements of temperature are not presently recommendable, but tympanic measurement performed by devices using the same technology as in this study is useful for screening purposes, especially among children aged 6 months to 5 years. For exact measurements of temperature, we still recommend the rectal method.

## Additional file

Additional file 1: Sample size calculation. (PDF 448 kb)
