# Supplementary material for: Forehead or ear temperature measurement cannot replace rectal measurements, except for screening purposes
Source: BMC Pediatr. 2018 Jan 26;18:15. doi: 10.1186/s12887-018-0994-1 (PMC5787302; doi:10.1186/s12887-018-0994-1)

Additional file.

Sample size calculation:

We used http://www.openepi.com/SampleSize/SSMean.htm

The calculation assumes normal distribution.

We assumed that the standard deviation of temperature measurements was 0.65 °C in accordance with formerly published results. https://www.ncbi.nlm.nih.gov/pubmed/15502654

We aimed at a sample size of minimum 99 children within each of the six age groups to be able to detect a minimum mean difference of +/- 0.3 °C with a standard deviation of 0.65 °C between the rectal and the alternative measurement, a 90% power, and a significance level of 0.05.

Calculation:

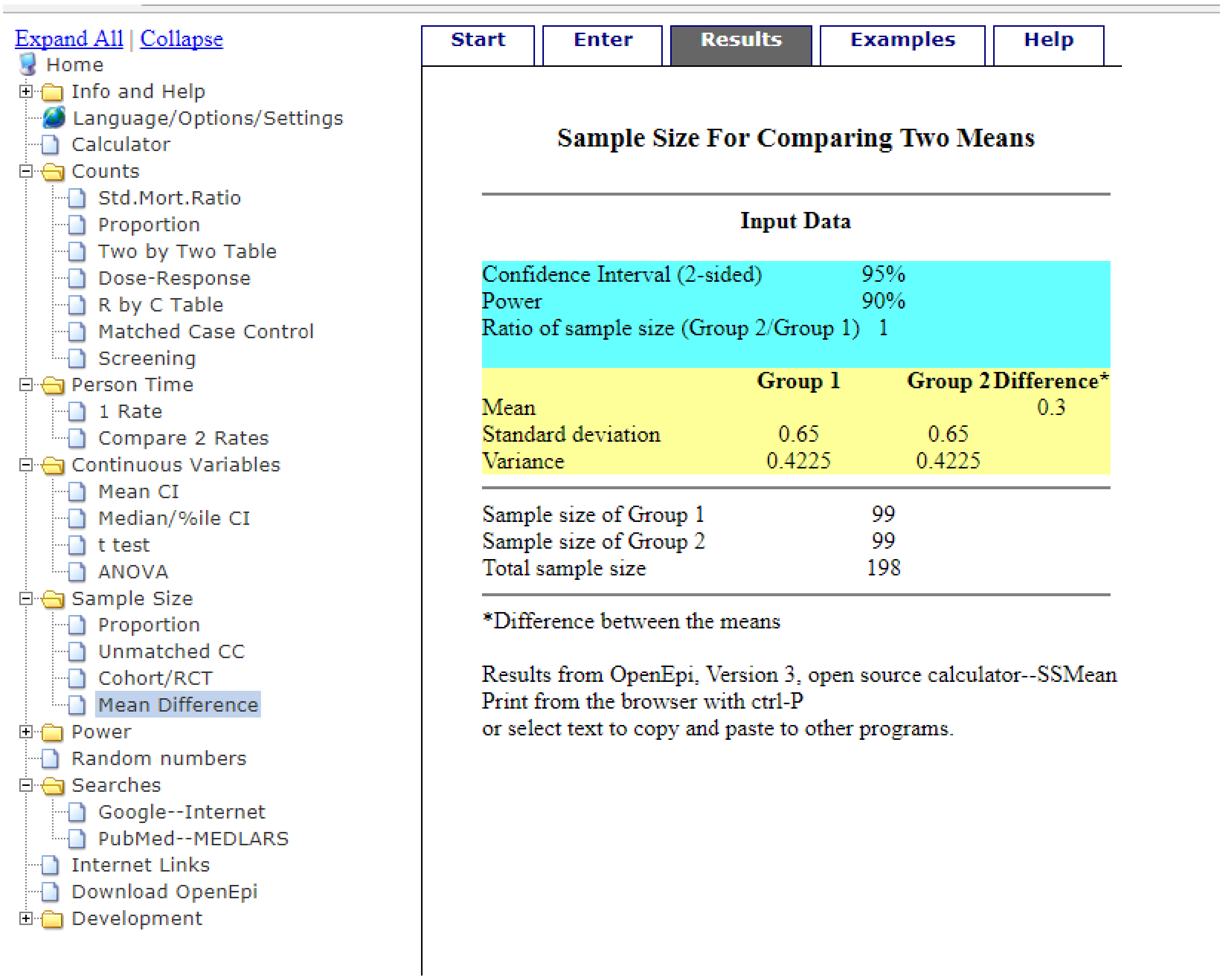

Our results of the study confirmed the assumption of normal distribution within all groups of children.

We used STATA 14. Qnorm function to assess this:

Total cohort:

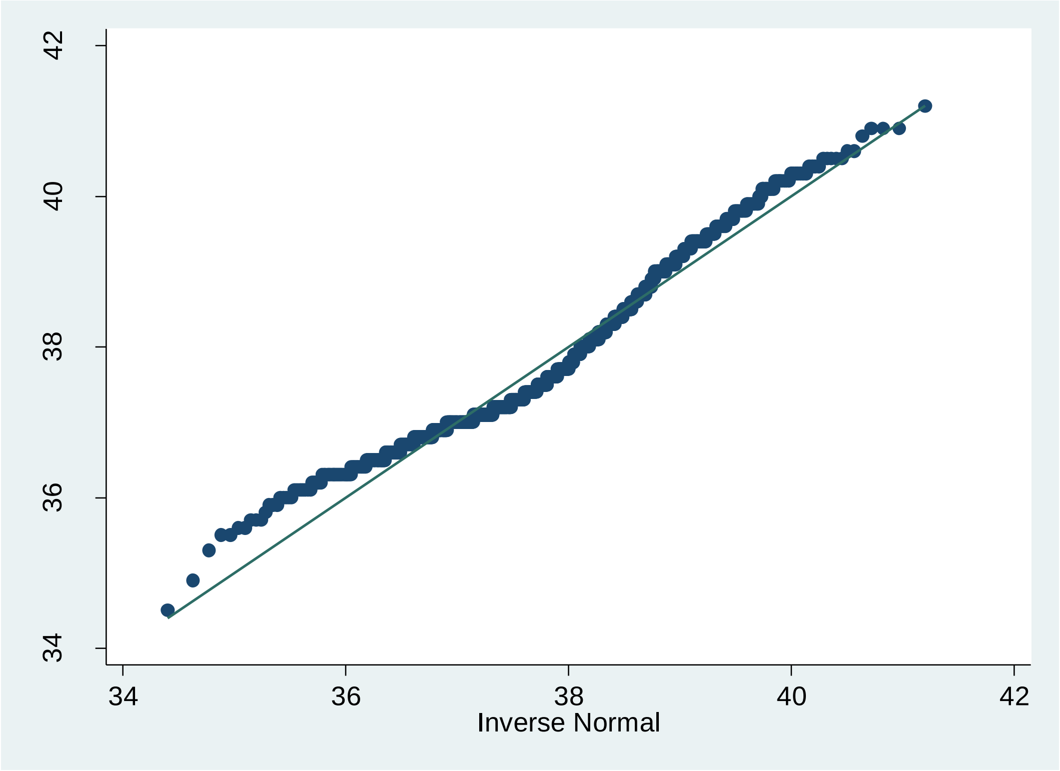

Agegroup

0-5 months

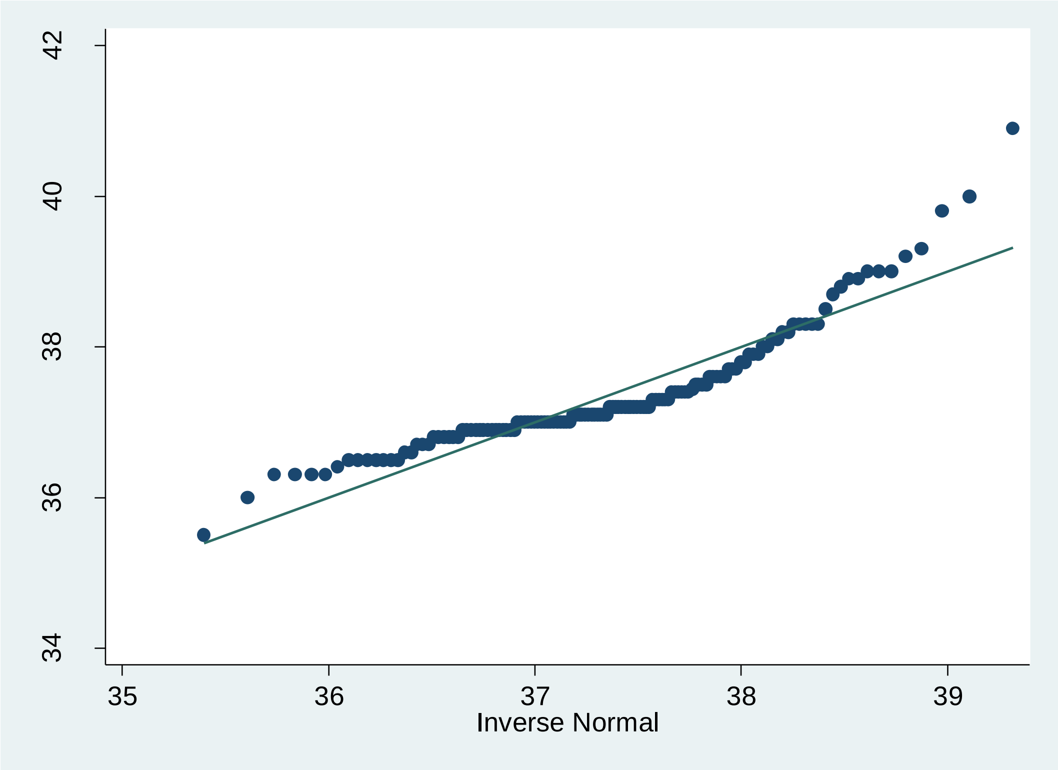

6-11 months

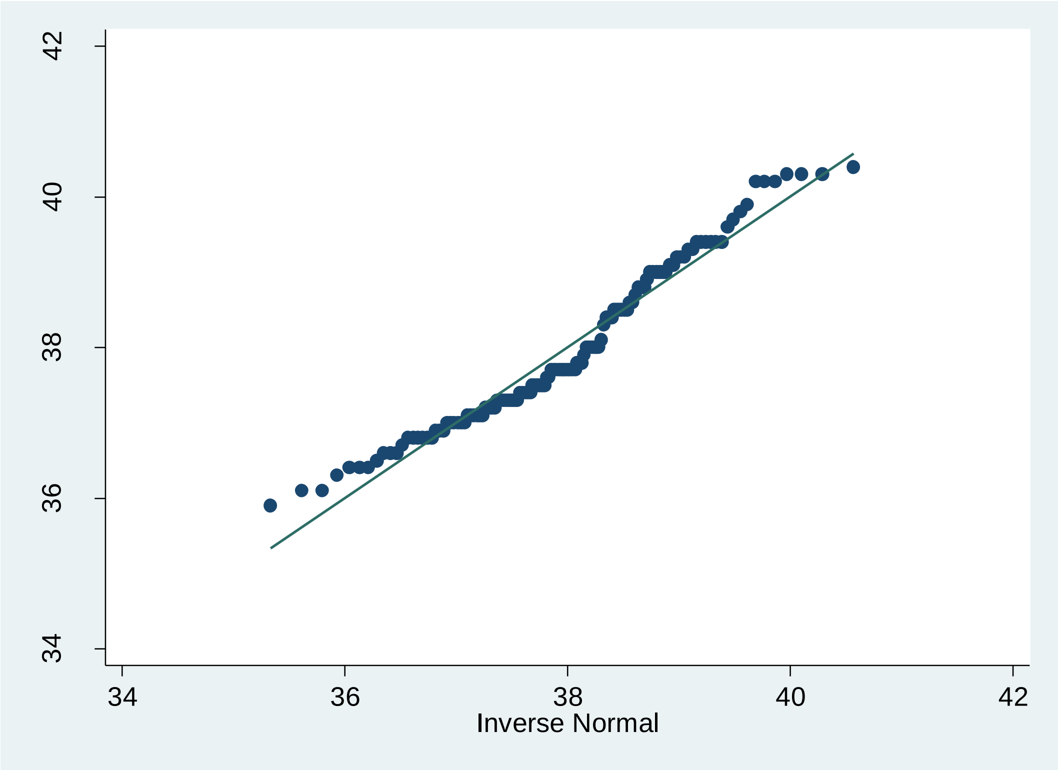

12-35 months

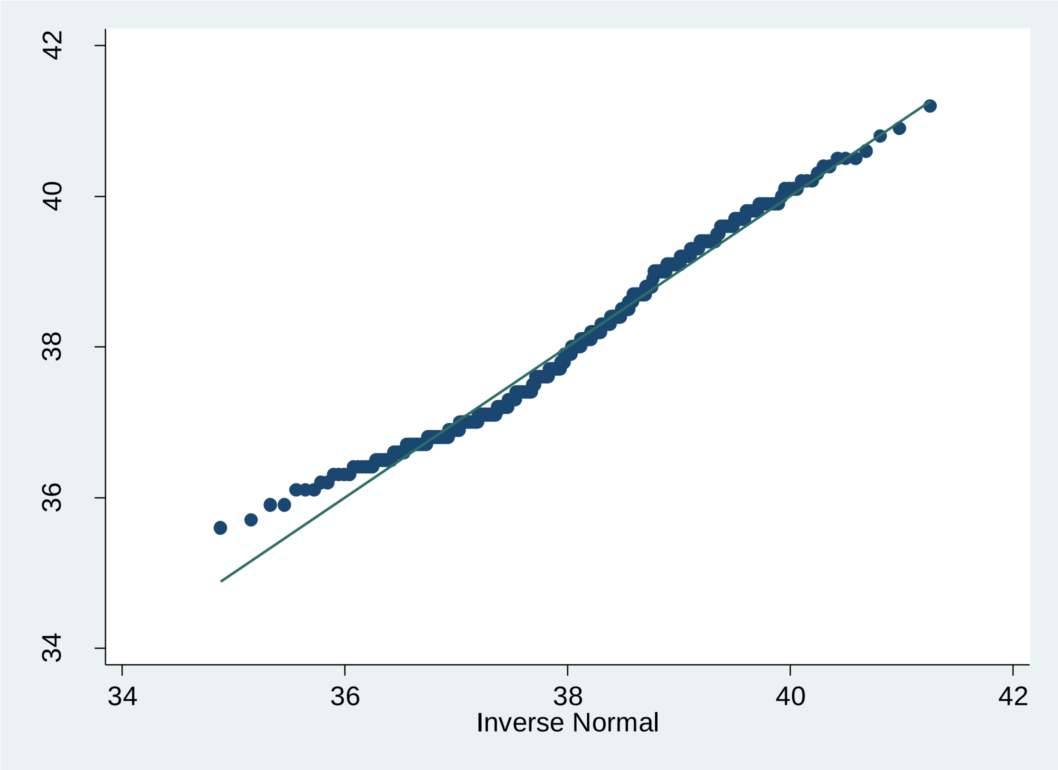

3-5 years

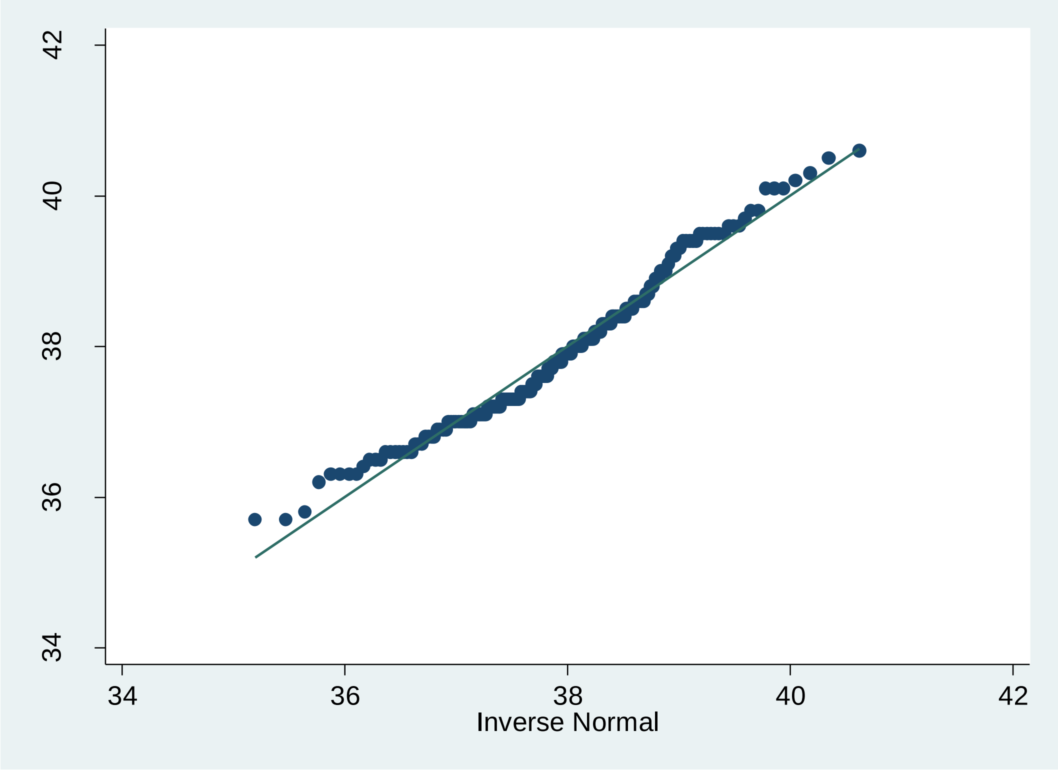

6-11 years

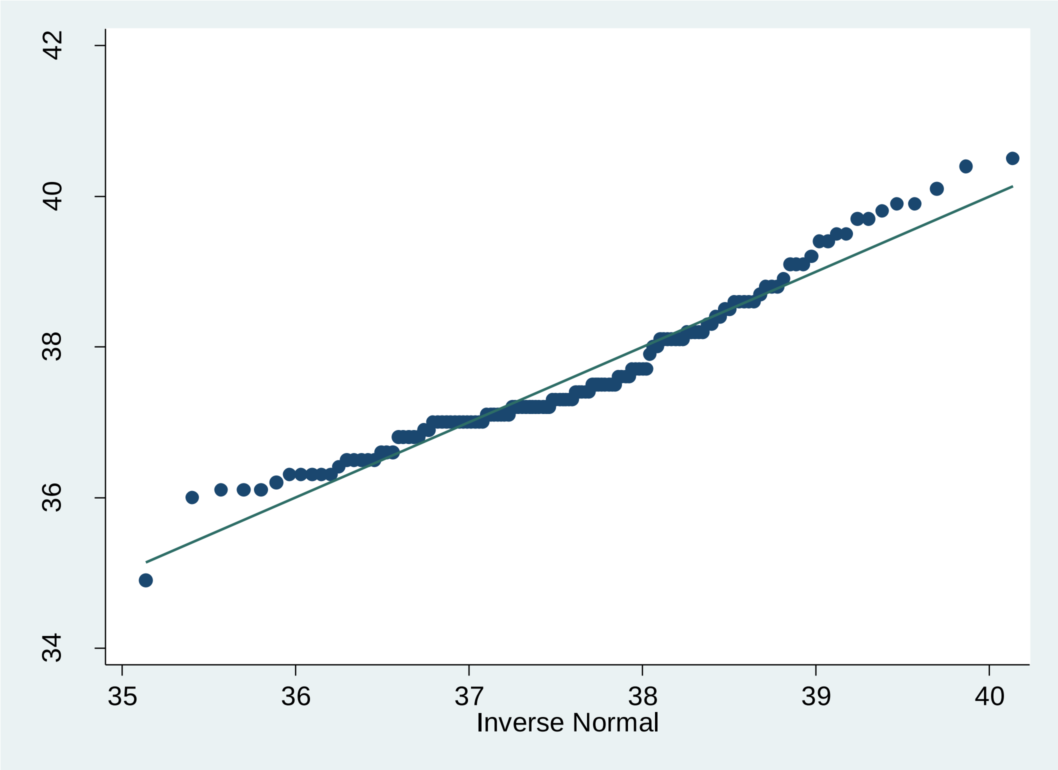

12-18 years

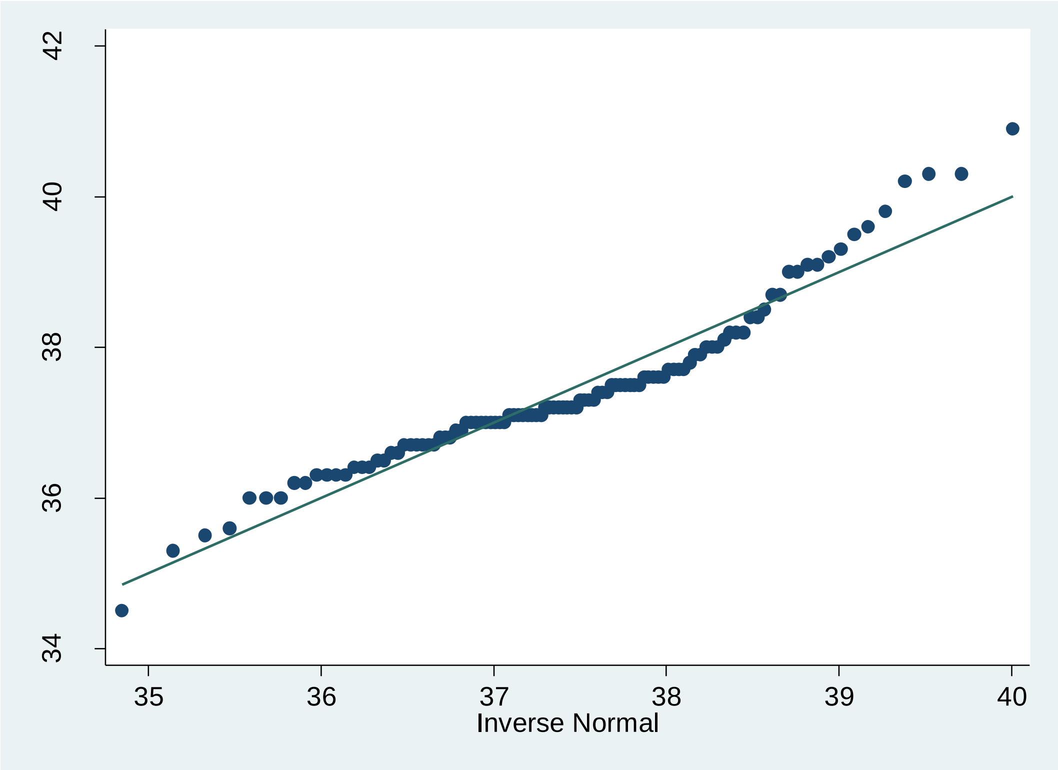

We assumed a SD of 0.6 °C.

Our results showed a SD of around 1.0 for the different age groups:

```
. sum tprectal if agegrp == 1

    Variable |        Obs        Mean    Std. Dev.       Min        Max
-------------+---------------------------------------------------------
    tprectal |        136    37.35618    .8024057       35.5       40.9

. sum tprectal if agegrp == 2

    Variable |        Obs        Mean    Std. Dev.       Min        Max
-------------+---------------------------------------------------------
    tprectal |        128    37.95234    1.082863       35.9       40.4

. sum tprectal if agegrp == 3

    Variable |        Obs        Mean    Std. Dev.       Min        Max
-------------+---------------------------------------------------------
    tprectal |        320    38.07356     1.16561       35.6       41.2

. sum tprectal if agegrp == 4

    Variable |        Obs        Mean    Std. Dev.       Min        Max
-------------+---------------------------------------------------------
    tprectal |        169    37.91006    1.077124       35.7       40.6

. sum tprectal if agegrp == 5

    Variable |        Obs        Mean    Std. Dev.       Min        Max
-------------+---------------------------------------------------------
    tprectal |        135    37.63556    1.024438       34.9       40.5

. sum tprectal if agegrp == 6

    Variable |        Obs        Mean    Std. Dev.       Min        Max
-------------+---------------------------------------------------------
    tprectal |        107     37.4271    1.095322       34.5       40.9

.
. sum tpore if agegrp == 1

    Variable |        Obs        Mean    Std. Dev.       Min        Max
-------------+---------------------------------------------------------
       tpore |        136    37.21324    .7833271       35.8       40.5

. sum tpore if agegrp == 2

    Variable |        Obs        Mean    Std. Dev.       Min        Max
-------------+---------------------------------------------------------
       tpore |        128    37.95391    1.078122         36       40.9

. sum tpore if agegrp == 3

    Variable |        Obs        Mean    Std. Dev.       Min        Max
-------------+---------------------------------------------------------
       tpore |        320    38.08531    1.134692       35.5       41.1

. sum tpore if agegrp == 4

    Variable |        Obs        Mean    Std. Dev.       Min        Max
-------------+---------------------------------------------------------
       tpore |        169        38.1    1.030372         36       41.1

. sum tpore if agegrp == 5

    Variable |        Obs        Mean    Std. Dev.       Min        Max
-------------+---------------------------------------------------------
       tpore |        135    37.81481    .9723428         36         41

. sum tpore if agegrp == 6

    Variable |        Obs        Mean    Std. Dev.       Min        Max
-------------+---------------------------------------------------------
       tpore |        107     37.4757    .9783352         35       40.9

.
end of do-file

.
```

We conclude, that due to the higher standard deviation than expected, our study size where 104-169 children were included in the subgroups will only have a 90% power to detect significant differences higher than 0.4- 0.45 °C instead of 0.3 °C.

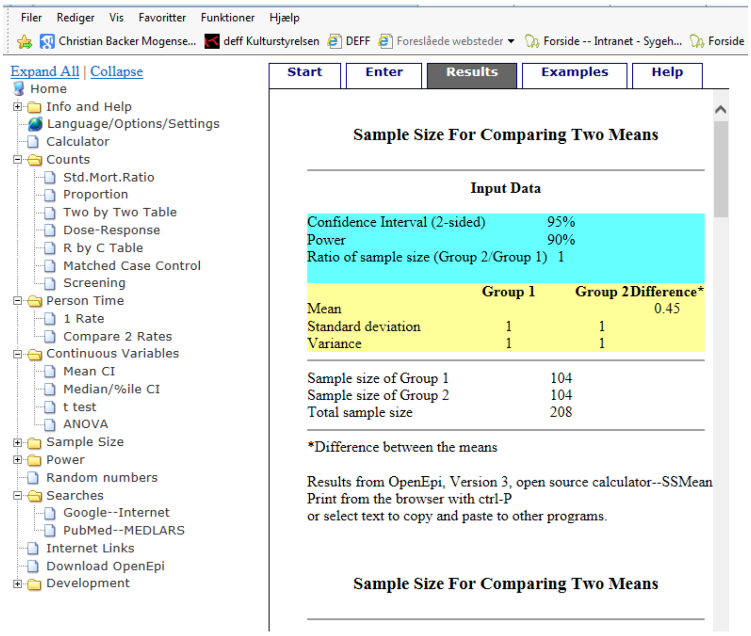

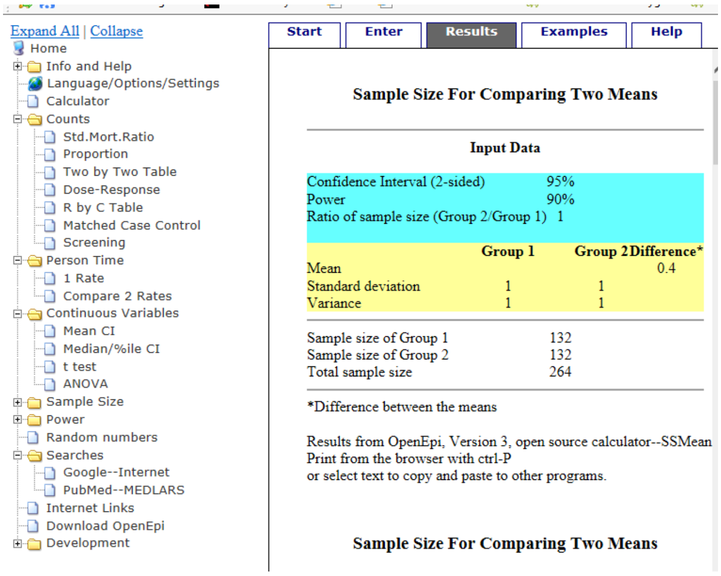

Supplement: Additional file 1: — Sample size calculation. (PDF 448 kb) [file 12887_2018_994_MOESM1_ESM.pdf]
